# Evaluation of a Luminex-Based Multiplex Immunoassay of Hemorrhagic Fever Viruses in Senegal

**DOI:** 10.1155/tbed/5529347

**Published:** 2025-01-20

**Authors:** Safietou Sankhe, Maryam Diarra, Mamadou Aliou Barry, Martin Faye, Cheikh Talla, Diogop Camara, Maimouna Mbanne, Pape Mbacke Sembene, Amadou Alpha Sall, Gamou Fall, Oumar Faye, Cheikh Loucoubar, Ousmane Faye, Ines Vigan-Womas, Keersten Michelle Ricks, Jessica Radzio-Basu, Moussa Moise Diagne

**Affiliations:** ^1^Virology Department, Institut Pasteur de Dakar, Dakar, Senegal; ^2^Animal Biology Department, Université Cheikh Anta Diop de Dakar, Dakar, Senegal; ^3^Epidemiology, Clinical Research and Data Science Department, Institut Pasteur de Dakar, Dakar, Senegal; ^4^Immunophysiopathology and Infectious Diseases Department, Institut Pasteur de Dakar, Dakar, Senegal; ^5^Diagnostic Systems Division, United States Army Medical Research Institute of Infectious Diseases, Fort Detrick, Frederick, Maryland, USA; ^6^Huck Institutes of the Life Sciences, Pennsylvania State University, University Park, Pennsylvania, USA

**Keywords:** ELISA, hemorrhagic fever viruses, MagPix, Senegal, serosurveillance, West Africa

## Abstract

Given the growing threat posed by viral hemorrhagic fevers, the development of surveillance tools is crucial to provide accurate and rapid solutions. Public health response involves risk assessment as well as effective and sustainable surveillance to ensure downstream communication and preparedness. A serological approach that offers high precision and throughput, cost efficiency, and multiplexing capacity is critical. In this work, we evaluated a Luminex-based multiplex microsphere immunoassay for five hemorrhagic fever viruses (HFVs) among the World Health Organization (WHO) blueprint. This five-plex MagPix immunoassay confirmed the presence of Rift Valley fever and Crimean–Congo hemorrhagic fever, but also revealed the exposure of human populations to hantaviruses in Senegal, underscoring the importance of regular serosurveillance in the identification of HFV hotspots.

## 1. Introduction

Over the last few decades, there has been a notable increase in the (re)emergence of viruses of major public health concern, particularly hemorrhagic fever viruses (HFVs) [1]. An increasingly recurrent hemorrhagic viral infection is the Ebola virus (EBOV) in the mononegavirales order and the Filoviridae family. Between 2017 and 2022, eight EBOV outbreaks were reported only in the Democratic Republic of the Congo [2]. The West African region faced the largest outbreak in history, with 11,325 fatal cases reported between 2013 and 2016 [3]. However, until now, only one confirmed EBOV-imported case was reported in Senegal in 2014 [4]. In addition to these pathogens potentially transmitted by bats, rodent-borne viruses such as the Lassa virus (LASV) or the Hantaviruses are also responsible for viral hemorrhagic fevers. LASV belongs to the Bunyavirales order and the Arenaviridae family and causes 500,000 cases and 10,000 fatalities annually in Nigeria and the Mano River region (Sierra Leone, Guinea, Liberia, and Côte d'Ivoire) [5, 6]. More recently, human infections have been reported in new countries such as Benin or Mali [7]. However, despite its major public health burden and the presence of the *Mastomys natalensis* reservoir; no human case has been identified in Senegal to date. The same situation is observed for Hantaviruses for which no human case has been detected to date in the sub-Saharan region despite evidence of circulating pathogenic species such as Seoul virus (SEOV) in Senegal in 2014 [8] and in Benin in 2018 [9].

Finally, the Crimean–Congo hemorrhagic fever (CCHFV) and Rift Valley fever (RVFV) viruses, two other Bunyavirales, are endemic arthropod-borne viruses in several African countries with numerous reported epizootic or outbreaks [10, 11]. In Senegal, viral amplification in vectors, sporadic cases, outbreaks, and evidence of exposure in the general population were recently observed [12–14].

Given the growing threat posed by these priority pathogens, the development of rapid response and surveillance tools is crucial to provide accurate and rapid solutions [15]. Population-based serosurveillance is a strategy that generates data to estimate and monitor infection trends, identify sociodemographic risk factors, and map disease geographical spread, enabling policymakers to make more informed and cost-effective decisions. A single plex enzyme-linked immunosorbent assay (ELISA) was previously used to assess the seroprevalence of CCHFV and RVFV in Senegal [14], with the potential for cross-reactivity and the need to repeat the test for each target. A serological approach that offers superior precision, dynamic range, high throughput, miniaturization, cost-efficiency, and multiplexing capacity is critical for sustainable HFV serosurveillance. A multiplex microsphere immunoassay targeting the immunoglobuline G antibody (IgG) antibodies against the different HFVs mentioned above has already been described [16].

In this paper, we describe the evaluation of this multiplex immunoassay using archived samples from Senegal.

## 2. Materials and Methods

### 2.1. Sampling

This study was carried out using two set of samples collected between February 2019 and March 2022: i) 88 human sera collected as part of a national seroprevalence study [14] and ii) 166 human sera from suspected cases of viral hemorrhagic fever obtained through national surveillance [17]. The first category of samples was obtained through a nationwide investigation of healthy people where whole blood was taken in the different households, while the second category of samples comes from febrile patients for whom blood was collected in healthcare facilities before shipping to the Institut Pasteur de Dakar (IPD) for laboratory analysis.

### 2.2. MagPix IgG Detection Assay

In this study, we screen the described human sera using a previously developed, qualitative multiplex magnetic beads–based assay [18] in which virus-specific sets of microspheres were combined into a multiplex assay that could detect nucleocapsid (NC)-targeted IgG in a single sample well (Table 1). These assays have been verified to be specific to each target using available animal models and known IgG-positive human samples [16, 19, 20]. The panel used five targeted HFVs including LASV, CCHFV, RVFV, EBOV, and Hantaan orthohantavirus (HTNV) following a protocol similar to what has been published [16].

Briefly, samples were inactivated for 30 min at 37°C and diluted 1:100 in PBST-SK (phosphate-buffered saline with 0.02% Tween 20 and 5% skim milk). The magnetic capture beads mix was diluted (1:250) in PBST (phosphate-buffered saline with 0.02% Tween 20). The diluted samples were added to a white 96-well plate and placed on the Luminex Plate Magnet. After a minute, the plate was discarded and the diluted samples, along with positive and negative controls, were added. These controls are a pool of known IgG positive human convalescent serum or known negative American human serum. The plate was covered with a plate sealer, shaken for 1 h, and then placed on the magnet for 60 s. The beads were washed with PBST three times. Human anti-IgG-PE was diluted (1:100) in PBST-SK, added to each well, and incubated at room temperature for 1 h. The beads were washed with PBST and 100 μL of PBST was added to the wells prior to reading on the MAGPIX instrument. The samples were assigned as positive for a given target when the signal over the negative control was greater than 20. This cutoff point was established based on a comparison of MagPix signal to noise ratios to microneutralization assays utilizing live virus on and independent Ghanian sample set (manuscript in preparation).

### 2.3. Indirect IgG ELISA

To evaluate the MagPix IgG detection assay, a subset of samples (88 sera samples from the national seroprevalence study) was characterized using a single plex indirect ELISA targeting IgG antibodies against RVFV and CCHFV, with viral antigens and in house mouse hyperimmune mouse ascitic fluids (HMAFs) stored in Virology Department biobank, and routinely used in the World Health Organization (WHO) Collaboration Center for Arboviruses and HFVs at IPD [14]. In-house reagents production was done as previously described [21], with mouse brain antigens produced by intracerebral inoculation of live viral strains, followed by brain collection after 6 days, homogenization, and inactivation with *β*-propiolactone, while HMAF were prepared by intraperitoneal inoculation of inactivated viral strains in adult Swiss Webster mice, followed by immunization with TG180 murine sarcoma cells, with fluid collection occurring 42 days later.

Briefly, standard ELISA plates (Immulon II 96-well microtiter plates; Dynatech laboratories, Inc., El Paso, TX, USA) were coated with the house-prepared HMAF specific for either virus diluted (1:1000) in PBST-SK. After washing, specific mouse brain antigens were diluted (1:100) in PBST-SK and incubated at 37° C for 1 h. A negative antigen control was added for each sample. After 1 h, the plate wells were washed three times with PBST-SK before adding the samples. Horseradish peroxidase conjugated goat anti-human IgG (Seracare, Milford, MA, USA) was added to the plates for 1 h of incubation. Specific binding was revealed by adding 3,3′, 5,5′-tetramethylbenzidine (TMB) and stopping with 2 N sulfuric acid (H_2_SO_4_). The plates were read on a 450 nm wavelength spectrophotometer and 620 nm as passive reference. Sera were considered positive when the difference in optical density between specific and nonspecific antigens was less than 0.20 and the ratio between the sample and the negative control was greater than 2.

## 3. Results

### 3.1. Validation of HFVs-MagPix IgG Detection Using Previously Characterized Human Samples

**Figure 1:**
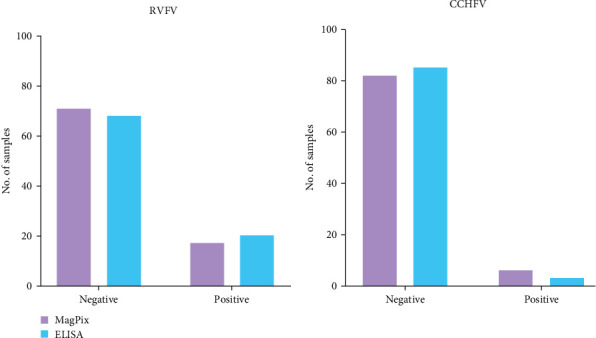
Comparison between the MagPix assay and the indirect enzyme-linked immunosorbent assay (ELISA) used by a World Health Organization (WHO) Collaborating Center for Arboviruses and hemorrhagic fever viruses (HFVs) for the detection of immunoglobuline G antibodies (IgGs). A panel of known IgG negative and IgG positive human samples for Rift Valley fever virus (RVFV) and Crimean–Congo hemorrhagic fever virus (CCHFV) was used for the evaluation. A cutoff point of 0.2 and 20 was chosen, respectively, for the indirect ELISA and the MagPix assay.

We tested 88 serum samples from a previous national seroprevalence study for IgG antibodies against arboviruses, specifically RVFV and CCHFV. These samples had been previously tested for antibodies against arboviruses and HFVs using indirect IgG ELISA as described above, with results available for comparison. The MagPix assay identified 17 positive samples for RVFV and 6 for CCHFV, while the indirect ELISA detected 20 and three positive samples, respectively, out of the 88 tested (Figure 1). The results obtained from both approaches were comparable with 19.3% versus 22.7% (*p*=0.496, Fisher's exact test) and 3.4% versus 6.8% (*p*=0.711, Fisher's exact test), respectively, for RVFV and CCHFV. The optical densities obtained for each sample using both methods are summarized in Supporting Information S1.

### 3.2. Prevalence of Selected HFVs Obtained From Human Samples in Senegal

**Figure 2:**
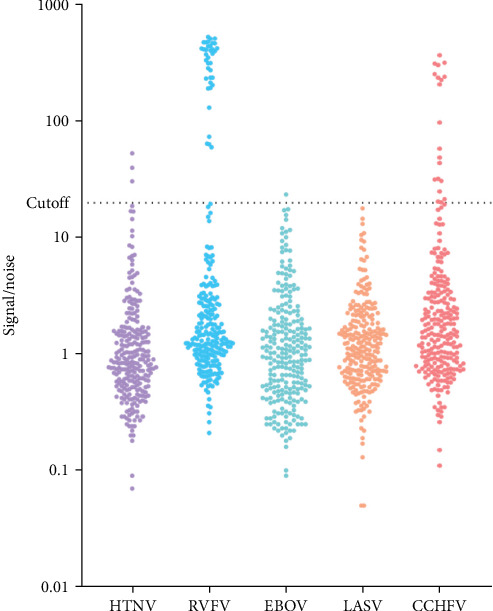
Distribution of immunoglobuline G antibody (IgG) responses to the five viruses tested by the MagPix panel of hemorrhagic fever viruses (HFVs). Samples collected from two different studies were evaluated for nucleocapsid (NC)-specific IgG responses using a signal-to-noise ratio. The cutoff point is 20.

After validating the MagPix multiplex HFV assay, 254 archived asymptomatic human sera, collected through a previous national Senegalese seroprevalence study, were analyzed directly using the MagPix assay without prior testing for IgM antibodies by ELISA. The MagPix assay detected antibody responses to all targeted HFVs except LASV. The lowest antibody levels were observed for EBOV (less than 1%, *n* = 1) and HTNV (1%, *n* = 3), while the highest seroprevalence was noted for CCHFV (8%, *n* = 20) and RVFV (15%, *n* = 37; Figure 2).

## 4. Discussion

We compared the data obtained from two serological approaches for the detection of HFV-specific IgG; a routinely used RVFV and CCHFV single-plex IgG ELISA and an NC-targeted multiplex MagPix assay. The results obtained on a panel of well-characterized human sera showed no significant difference between the two methods, even if some IgG positive ELISA samples with borderline optical density were not detected by the MagPix approach. Indeed, it was already noted than multiplex screening typically has lower sensitivity limits compared to individual target detection [22]. Additionally, the ELISA detects a more polyclonal response to RVFV and CCHFV by the use of HMAFs and inactivated viral preparations compared to the MagPix assay to detect the response to a specific antigen (i.e., antinucleoprotein response for CCHFV or RVFV). We acknowledge that the sample size in this study was limited, reflecting its design as a pilot to assess the feasibility and utility of the multiplex MagPix platform. While the results offer valuable insights into the concordance between the two serological methods, the small sample size may limit the broader applicability of these findings. Further studies with larger cohorts will be essential to validate these results and enhance the interpretation and reliability of multiplex assays for HFV serosurveillance.

Despite these limitations, the results validated the use of the MagPix multiplex HFV assay on an additional set of 254 archived asymptomatic human sera collected through a previous national Senegalese seroprevalence study and routine surveillance of febrile diseases. The screening showed reactivity for all targeted HFVs with a greater exposition to RVFV and CCHFV. The observed anti-RVFV and anti-CCHFV IgG levels were higher compared to the last study of seroprevalence done in Senegal in 2020 [14], where the crude seroprevalences were 3.94% and 0.7%, respectively. However, the target populations in both studies were different, biasing the comparison, with 2020 observations based on a designed study of SARS-CoV-2 and dengue seroprevalence.

None of the samples exhibited LASV reactivity above the determined cutoff point. This is in line with a previous study published in 1988 in which a low seroprevalence for LASV was detected in rodents in Senegal with no evidence of seroconversion in populations in the eastern and western parts of the country [23]. Therefore, the low antibody titers suggested a potential detection of another arenavirus heterologous to LASV. However, the recent emergence of the virus in other countries in West Africa [24–27] highlights that the spatial distribution of the virus and its reservoirs is expanding and that continued surveillance is critical.

Similar to LASV, a low seroprevalence of anti-EBOV and anti-HTNV IgG antibodies was found in the sample set. Only one sample showed low positive reactivity to the EBOV antigen. This result highlights a potential exposure to EBOV or a related filovirus in Senegal as previously described in Mali, where few imported cases were reported [16]. It would be tempting to present the scenario of possible immunological sequelae after the detection of a single imported EBOV case in Senegal in 2014, but this remains unlikely given that no transmission chain or secondary cases had been identified at that time [4]. However, a study of the seropositivity of EBOV antibodies in Sierra Leone residents revealed an 8% positivity among those who never experienced symptoms, highlighting the impact of the 2014–2016 outbreak [28]. Serological evidence of EBOV exposure in rural Guinea before the 2014 West African Epidemic was also reported [29], suggesting that the natural history of EBOV and other filoviruses in West Africa requires further investigation.

Three samples (1%) exhibited anti-HTNV IgG reactivity in the total study population. This is less than the seroprevalences of hantavirus observed in Côte d'Ivoire and the Democratic Republic of the Congo [30], as well as Ethiopia [31], but comparable to Guinea [32] and South Africa [33]. SEOV, a viral species within the *Orthohantavirus* genus of rodent-borne viruses, has been identified in black rats in Senegal [8], however, no additional studies have been performed since then. Our study provides new evidence of the circulation of hantavirus in Senegal with the first serological identification made in human samples. Furthermore, the prevalence of Hantavirus could be underestimated in Senegal, since no surveillance program has been established. For example, one of the highest seroprevalences in Africa was found in Sudan with 10% IgG positive hantaviruses through end-stage renal disease patients surveillance [34]. Previous studies showed that sociocultural, ecological and consumption factors impact exposure risks [35, 36], highlighting the need to have a specific surveillance system for hantaviruses.

## 5. Conclusions

Our study demonstrated the usefulness of implementing a MagPix-based multiplex IgG detection assays for HFVs in Senegal. Moreover, this approach is financially more affordable since a single reaction allows multiple detection while preserving biological material. Public health responses involve risk assessment and effective and sustainable surveillance to ensure downstream communication and preparedness. In our work, the described multiplex microsphere immunoassay showed good concordance with assays used in a WHO Collaboration Center for Arboviruses and HFVs regarding the previously identified RVFV and CCHFV human samples in Senegal. Furthermore, evidence of exposure to other highly pathogenic zoonotic viruses, such as EBOV and HNTV, or to associated viruses was found. In general, this study emphasized the importance of regular serosurveillance in Senegal and the region with the potential to identify potential hotspots for HFVs.

## Figures and Tables

**Table 1:** Targets included in the MagPix HFVs assay.

Agent	*⁣* ^*∗*^Antigen	IgG positive control
Hantaan (HTNV)	NP	HTNV IgG + sera
EBOV	NP	EBOV IgG + sera
CCHFV	NP	CCHFV IgG + sera
LASV	NP	LASV IgG + sera
RVFV	NP	RVFV IgG + sera

Abbreviations: CCHFV, Crimean–Congo hemorrhagic fever virus; EBOV, Ebola virus; HFV, hemorrhagic fever virus; HTNV, Hantaan orthohantavirus; IgG, immunoglobuline G antibody; LASV, Lassa virus; RVFV, Rift Valley fever virus.

*⁣* ^*∗*^All antigens commercially available from Native Antigen Company.

## Data Availability

All data supporting the conclusions of this study are available in the manuscript and the Supporting Information.

## Supporting Information

Supporting Information Supporting Information S1. Signal-to-noise ratio for samples evaluated using the indirect ELISA and MagPix assay.
