# Supplementary material for: Evaluation of a Luminex-Based Multiplex Immunoassay of Hemorrhagic Fever Viruses in Senegal
Source: Transbound Emerg Dis. 2025 Jan 20;2025:5529347. doi: 10.1155/tbed/5529347 (PMC12016757; doi:10.1155/tbed/5529347)
Supplement: Supporting Information — S1. Signal-to-noise ratio for samples evaluated using the indirect ELISA and MagPix assay. [file 5529347.f1.docx]

**Supplementary S1.** Signal-to-noise ratio for samples evaluated using the indirect ELISA and MagPix assay.

|  | **MagPix IgG DETECTION** | | **INDIRECT ELISA IgG** | |
| --- | --- | --- | --- | --- |
| **SAMPLE** | **RVF** | **CCHF** | **RVF** | **CCHF** |
| 357759 | 1,83 | 4,04 | 0,024 | 0,018 |
| 357760 | 1,80 | 4,27 | 0,016 | 0,04 |
| 357761 | 1,56 | 6,79 | 0,092 | 0,034 |
| 357762 | 0,82 | 1,27 | 0,101 | 0,031 |
| 357763 | 1,48 | 1,24 | 0,015 | 0,023 |
| 357764 | 3,83 | 2,70 | 0,002 | 0,056 |
| 357765 | 2,14 | 0,49 | 0,008 | 0,033 |
| 357766 | 1,79 | 0,84 | 0,058 | 0,011 |
| 357767 | 3,32 | 6,85 | 0,04 | 0,022 |
| 357768 | 4,59 | 3,03 | 0,041 | 0,014 |
| 357769 | 2,23 | 3,38 | 0,054 | 0,059 |
| 357770 | 8,39 | 4,03 | 0,022 | 0,038 |
| 357800 | 1,72 | 1,03 | 0,022 | 0,011 |
| 357801 | 1,79 | 7,47 | 0 | 0,001 |
| 357802 | 5,39 | 2,51 | 0,003 | 0,006 |
| 357803 | 4,08 | 1,46 | 0,016 | 0,002 |
| 357804 | 73,95 | 43,89 | 0,027 | 0,027 |
| 357842 | 0,56 | 0,67 | 0,019 | 0,007 |
| 357844 | 0,58 | 0,33 | 0,017 | 0,004 |
| 357845 | 0,69 | 1,64 | 0,004 | 0,006 |
| 357856 | 2,15 | 0,71 | 0,022 | 0,005 |
| 357858 | 1,05 | 0,88 | 0,006 | 0,016 |
| 357859 | 2,71 | 1,97 | 0,044 | 0,003 |
| 357860 | 1,04 | 1,82 | 0,038 | 0,006 |
| 357861 | 0,81 | 0,68 | 0 | 0,01 |
| 357862 | 0,79 | 2,77 | 0 | 0,041 |
| 357863 | 0,80 | 9,43 | 0,013 | 0,005 |
| 357864 | 2,85 | 3,58 | 0,017 | 0,005 |
| 357865 | 3,92 | 2,38 | 0,004 | 0,019 |
| 357866 | 1,06 | 0,70 | 0,004 | 0,024 |
| 357868 | 1,23 | 2,35 | 0,016 | 0,005 |
| 357869 | 1,21 | 1,43 | 0,011 | 0,007 |
| 357969 | 1,37 | 1,24 | 0,021 | 0,009 |
| 357970 | 0,59 | 0,26 | 0,009 | 0,006 |
| 357997 | 0,53 | 0,94 | 0,009 | 0,038 |
| 357998 | 0,47 | 0,77 | 0,073 | 0,015 |
| 358000 | 2,70 | 1,39 | 0,038 | 0,024 |
| 358001 | 0,92 | 0,58 | 0,062 | 0,02 |
| 358002 | 0,95 | 2,00 | 0,011 | 0,038 |
| 358004 | 1,34 | 1,14 | 0,021 | 0,013 |
| 358005 | 1,12 | 32,10 | 0,013 | 0,065 |
| 358006 | 1,24 | 1,42 | 0,046 | 0,035 |
| 358007 | 0,75 | 0,69 | 0,018 | 0,029 |
| 358008 | 0,71 | 2,16 | 0,095 | 0,012 |
| 358010 | 1,24 | 20,45 | 0,035 | 0,059 |
| 358103 | 0,68 | 30,73 | 0,019 | 0,22 |
| 358309 | 2,10 | 1,26 | 0,05 | 0,003 |
| 358310 | 0,57 | 1,46 | 0,012 | 0,003 |
| 358311 | 0,65 | 0,61 | 0,021 | 0,001 |
| 358312 | 1,16 | 1,02 | 0,028 | 0,007 |
| 358330 | 1,21 | 1,07 | 0,017 | 0,003 |
| 358331 | 0,97 | 0,72 | 0,086 | 0,019 |
| 358333 | 1,10 | 0,75 | 0,004 | 0,004 |
| 358334 | 1,14 | 5,47 | 0,032 | 0,011 |
| 358339 | 1,55 | 1,82 | 0 | 0 |
| 358673 | 313,32 | 2,49 | 1,10 | 0 |
| 358718 | 474,57 | 8,14 | 0,53 | 0 |
| 358722 | 479,00 | 2,22 | 0,84 | 0,018 |
| 358723 | 412,68 | 12,98 | 0,79 | 0 |
| 358726 | 402,55 | 3,01 | 0,74 | 0,002 |
| 358727 | 189,96 | 10,90 | 0,43 | 0,009 |
| 358728 | 464,11 | 5,39 | 0,62 | 0 |
| 358731 | 437,53 | 2,24 | 0,71 | 0,002 |
| 358732 | 0,67 | 1,05 | 0,001 | 0,001 |
| 358734 | 0,94 | 0,11 | 0,67 | 0,003 |
| 358738 | 508,65 | 31,70 | 0,66 | 0,005 |
| 358760 | 272,74 | 3,82 | 0,33 | 0,006 |
| 358761 | 1,22 | 3,90 | 0,59 | 0,68 |
| 358763 | 509,94 | 4,17 | 0,65 | 0,006 |
| 358807 | 526,55 | 4,40 | 0,80 | 0,004 |
| 358817 | 474,19 | 4,65 | 1,03 | 0,006 |
| 358934 | 419,10 | 3,23 | 1,23 | 0,021 |
| 358958 | 467,47 | 2,07 | 1,29 | 0,012 |
| 358991 | 1,34 | 1,46 | 0,008 | 0,045 |
| 358992 | 1,16 | 0,74 | 0,026 | 0,018 |
| 358993 | 1,29 | 1,02 | 0,03 | 0,005 |
| 358994 | 1,23 | 0,65 | 0,313 | 0,016 |
| 358995 | 16,33 | 1,38 | 0,001 | 0,013 |
| 358996 | 0,80 | 0,79 | 0,023 | 0,015 |
| 358997 | 0,70 | 7,37 | 0,047 | 0,035 |
| 358998 | 0,99 | 2,17 | 0,06 | 0,015 |
| 358999 | 0,53 | 0,66 | 0,036 | 0,003 |
| 359004 | 1,10 | 2,31 | 0,008 | 0,017 |
| 359005 | 1,00 | 1,51 | 0,043 | 0,012 |
| 359012 | 6,79 | 0,63 | 0,012 | 0,07 |
| 359024 | 1,55 | 366,58 | 0,003 | 1,12 |
| 359132 | 440,36 | 4,43 | 0,80 | 0,007 |
| 359151 | 2,04 | 0,79 | 0,76 | 0,019 |
| Total positive samples* | 17 | 6 | 20 | 3 |

* Positive samples were determined for the MAGPIX assay using a cut-off of 20 and for the ELISA using a cut-off of 0.2.
